# Resurgence of SARS-CoV-2: Detection by community viral surveillance

**DOI:** 10.1126/science.abf0874

**Published:** 2021-04-23

**Authors:** Steven Riley, Kylie E. C. Ainslie, Oliver Eales, Caroline E. Walters, Haowei Wang, Christina Atchison, Claudio Fronterre, Peter J. Diggle, Deborah Ashby, Christl A. Donnelly, Graham Cooke, Wendy Barclay, Helen Ward, Ara Darzi, Paul Elliott

**Affiliations:** 1 School of Public Health, Imperial College London, London, UK.; 2 MRC Centre for Global Infectious Disease Analysis and Abdul Latif Jameel Institute for Disease and Emergency Analytics, Imperial College London, London, UK.; 3 Centre for Health Informatics, Computing, and Statistics (CHICAS), Lancaster Medical School, Lancaster University, Lancaster, UK.; 4 Health Data Research UK, London, UK.; 5 Department of Statistics, University of Oxford, Oxford, UK.; 6 Department of Infectious Disease, Imperial College London, London, UK.; 7 Imperial College Healthcare NHS Trust, London, UK.; 8 National Institute for Health Research Imperial Biomedical Research Centre, London, UK.; 9 Institute of Global Health Innovation, Imperial College London, London, UK.; 10 MRC Centre for Environment and Health, School of Public Health, Imperial College London, London, UK.

## Abstract

Surveillance of the severe acute respiratory syndrome coronavirus 2 (SARS-CoV-2) pandemic has mainly relied on case reporting, which is biased by health service performance, test availability, and test-seeking behaviors. We report a community-wide national representative surveillance program in England based on self-administered swab results from ~594,000 individuals tested for SARS-CoV-2, regardless of symptoms, between May and the beginning of September 2020. The epidemic declined between May and July 2020 but then increased gradually from mid-August, accelerating into early September 2020 at the start of the second wave. When compared with cases detected through routine surveillance, we report here a longer period of decline and a younger age distribution. Representative community sampling for SARS-CoV-2 can substantially improve situational awareness and feed into the public health response even at low prevalence.

## Community virus surveillance

Even highly effective vaccines will not save us from the need to monitor severe acute respiratory syndrome coronavirus 2 (SARS-CoV-2) activity, perhaps for years to come. Public health institutions will need early warning of any uptick in cases to prepare and deploy interventions as required. Riley *et al.* developed a community-wide program that was designed to detect resurgence at low prevalence and has been used to track SARS-CoV-2 virus across England. In the four rounds of sampling from May to September 2020, almost 600,000 people representative of all communities were monitored. The results revealed the greatest prevalence among 18- to 24-year-olds, with increasing incidence among older age groups and elevated odds of infection among some communities. This testing approach offers a model for the type of real-time, country-wide population-based surveillance work that needs to be conducted to monitor SARS-CoV-2.

*Science*, abf0874, this issue p. 990

## Teaser

Representative community antigen sampling can improve situational awareness and help evaluate interventions at low SARS-CoV-2 prevalence.

Ahead of widespread rollout of effective vaccines in most countries (*1*–*3*), severe acute respiratory syndrome coronavirus 2 (SARS-CoV-2) infection continues to cause substantial COVID-19 morbidity and mortality globally (*4*). As variants with potentially increased transmissibility emerge (*5*), populations around the world continue to trade off between social interactions and risk of infection (*6*). However, reduced social contact (*7*) has adverse effects on levels of economic activity (*8*), non-COVID-19–related health, and overall well-being (*9*). The ability of both individuals and governments to continue to balance these competing demands requires accurate and timely knowledge of the spread of the virus in the population so that informed choices about interventions can be made.

Data streams based on respiratory symptoms, such as those used for COVID-19 surveillance in most countries, are prone to biases that can obscure underlying trends, such as variations in test availability and test-seeking behavior (*10*). Some countries have augmented these systems with surveys of virus prevalence in the wider population, but these have mostly been one-off activities, for example, as in Wuhan, China (*11*), or were designed explicitly as interventions, for example, as in Slovakia (*12*). Here we show results from the Real-time Assessment of Community Transmission-1 (REACT-1) study, a representative community-wide program that is tracking prevalence of SARS-CoV-2 across England through repeated random population-based sampling (*13*). This program was designed to rapidly detect resurgence of SARS-CoV-2 transmission, including at low prevalence, thus providing early warning of any upturn in infections, which can help with policy response and enable timely implementation of public health interventions.

**Fig. 1:**
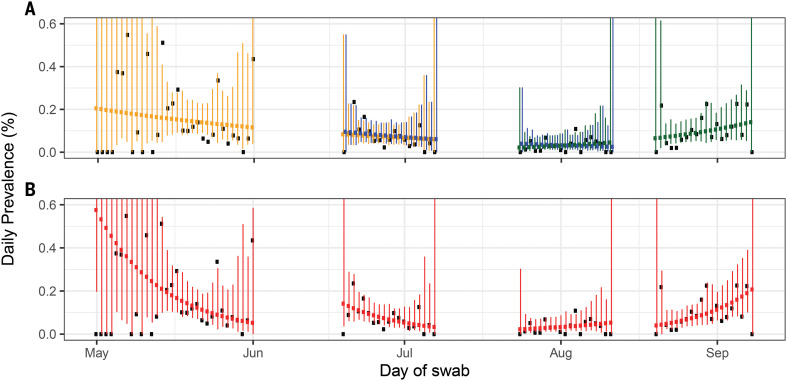
Constant growth rate models fit to REACT-1 data for sequential and individual rounds. (**A**) Model fits to REACT-1 data for sequential rounds 1 and 2 (yellow), 2 and 3 (blue), and 3 and 4 (green). Vertical lines show 95% prediction intervals for models. Black points show observations. See Table 1 for *R* estimates. (**B**) Models fit to individual rounds only (red). Note that only 585,004 of 596,965 tests had dates available and were included in the analysis (465 out of 473 positives were included).

Over the course of four rounds, from 1 May to 8 September 2020, we invited more than 2.4 million people to join the study, from whom we obtained ~596,000 tested swabs (Table 1) for an overall response rate of ~25% (table S1). Between round 1 (1 May to 1 June 2020) and round 2 (19 June to 7 July) there was a fall in weighted prevalence from 0.16% (95% confidence interval: 0.12%, 0.19%) to 0.088% (0.068%, 0.11%) (Table 1 and Fig. 1). Infections fell further, to their lowest observed value, in round 3 (24 July to 11 August), with 54 positive samples out of 161,560 swabs, giving a weighted prevalence of 0.040% (0.027%, 0.053%). In comparison, a 100-fold higher prevalence of ~5% was seen at the peak of the first UK wave, based on a daily incidence of infection in the UK of >300,000 (*14*) and assuming that individuals would test swab-positive for ~10 days on average (*15*). Prevalence then increased in round 4 (20 August to 8 September), where we found 137 positive samples out of 154,325 swabs, giving a weighted prevalence of 0.13% (0.10%, 0.15%).

**Table 1:** Unweighted and weighted prevalence (95% confidence interval) of swab positivity across four rounds of REACT-1.

**Parameter**	**Round 1**	**Round 2**	**Round 3**	**Round 4**
First sample	1 May 2020	19 June 2020	24 July 2020	20 August 2020
Last sample	1 June 2020	7 July 2020	11 August 2020	8 September 2020
Recruitment letters sent	395,000	600,000	710,000	710,000
Swabs sent	161,497	219,633	225,615	211,291
Tested swabs	120,620	159,199	161,560	154,325
Swab response rate	75%	72%	72%	73%
Overall response rate	31%	27%	23%	22%
Positive swabs	159	123	54	137
Unweighted prevalence (95% CI)	0.132% (0.113%, 0.154%)	0.077% (0.065%, 0.092%)	0.033% (0.025%, 0.043%)	0.089% (0.075%, 0.105%)
Weighted prevalence (95% CI)	0.156% (0.124%, 0.188%)	0.088% (0.068%, 0.109%)	0.040% (0.027%, 0.053%)	0.125% (0.096%, 0.154%)

Using a model of constant exponential growth and decay (*16*), we quantified this fall and rise in prevalence in terms of halving and doubling times and reproduction number *R* (Fig. 1 and Table 2). Over rounds 2 and 3 (19 June to 11 August), prevalence fell with an estimated halving time of 27 days (95% credible intervals: 20, 42) corresponding to an *R* value of 0.85 (0.79, 0.90). Prevalence then increased over rounds 3 and 4 (24 July to 8 September), with a doubling time of 17 (14, 23) days corresponding to an *R* value of 1.28 (1.20, 1.36). Our estimates of *R* and doubling times were similar in sensitivity analyses among nonsymptomatic people [average 72% (95% confidence interval: 67%, 76%)] or those positive for both the envelope protein (E) gene and nucleocapsid protein (N) gene (table S2).

**Table 2:** Fitted growth rates, reproduction numbers, and doubling times (95% credible intervals) for SARS-CoV-2 swab positivity in England.

**Data**	Round(s)^*^	**Number of** **participants** **or cases**	**Growth rate *r*** **(per day)**	***P* (*r* > 0)**	**Reproduction** **number**	**Doubling (+) or halving (−)** **time (days)**
REACT All	1	110,944	−0.077 (−0.107, −0.046)	<0.01	0.57 (0.44, 0.73)	−9.0 (−6.5, −14.9)
	2	157,428	−0.089 (−0.130, −0.032)	<0.01	0.52 (0.36, 0.81)	−7.8 (−5.3, −21.4)
	3	162,619	0.049 (−0.012, 0.109)	0.94	1.34 (0.93, 1.83)	14.2 (−58.6, 6.4)
	4	153,964	0.086 (0.050, 0.122)	>0.99	1.64 (1.35, 1.95)	8.0 (13.8, 5.7)
	1 and 2	268,422	−0.018 (−0.025, −0.012)	<0.01	0.89 (0.85, 0.92)	−37.9 (−28.0, −57.5)
	2 and 3	320,047	−0.025 (−0.034, −0.017)	<0.01	0.85 (0.79, 0.90)	−27.3 (−20.1, −41.6)
		3 and 4	316,583	0.041 (0.030, 0.051)	>0.99	1.28 (1.20, 1.36)	17.0 (22.8, 13.5)
Routine surveillance data	1	69,299	−0.034 (−0.042, −0.027)	<0.01	0.80 (0.75, 0.84)	−20.3 (−16.7, −26.0)
	2	11,523	−0.018 (−0.032, −0.004)	0.01	0.89 (0.81, 0.97)	−38.5 (−21.9, −164.1)
	3	15,172	0.026 (0.004, 0.049)	0.99	1.18 (1.03, 1.34)	26.2 (161.4, 14.2)
	4	31,209	0.085 (0.067, 0.104)	>0.99	1.63 (1.48, 1.79)	8.1 (10.3, 6.7)
	1 and 2	97,255	−0.029 (−0.031, −0.027)	<0.01	0.82 (0.81, 0.84)	−23.7 (−22.0, −25.7)
	2 and 3	36,393	0.007 (0.003, 0.012)	>0.99	1.05 (1.02, 1.07)	93.6 (207.6, 60.6)
	3 and 4	56,064	0.029 (0.023, 0.035)	>0.99	1.19 (1.15, 1.24)	24.1 (30.7, 20.1)

*See Table 1 for start and end dates of rounds

We compared epidemic trends estimated from the REACT-1 data above with those based on routine surveillance data (Fig. 1, figs. S1 and S2, and Table 2) over the same period. Numbers of routine surveillance cases were growing from the start of round 2 to the end of round 3 (19 June to 11 August), with a corresponding *R* of 1.05 (1.02, 1.07) (Table 2), when swab positivity was declining in REACT-1. *R* estimates from routine surveillance data were likely upwardly biased, because there was a near-doubling of test capacity during this period (*17*) (fig. S1). These findings are consistent with experience in the UK during the 2009 influenza pandemic, when there were substantial temporal variations in the sensitivity of case-based polymerase chain reaction surveillance (*18*).

We also observed an apparent shift from decline to growth using within-round data (fig. S3 and Table 2). During round 3 (24 July to 11 August), with 94% probability, the epidemic had started to grow with a doubling time of 14 days (95% credible interval: from halving every 59 days to doubling every 6.4 days), corresponding to an *R* of 1.34 (0.93, 1.83) (Fig. 1 and Table 1). During round 4 (20 August to 8 September), the doubling time decreased to 8.0 (5.7, 14) days, with an *R* of 1.64 (1.35, 1.95). In response to the rapidly increasing epidemic, the UK government announced a more stringent social distancing measure called the “rule of six,” prohibiting gatherings of more than six people (*19*).

We relaxed our assumption of constant growth or decay using a flexible p-spline (*16*) (fig. S1) and inferred a plateau or slight increase in prevalence in July 2020 in the gap between rounds 2 and 3. As a result, the prevalence for round 3 started higher than expected from the data observed at the end of round 2, a pattern similar to that seen in data from the Office for National Statistics Coronavirus (COVID-19) Infection Survey pilot (*20*). Using the p-spline, we estimated that lowest prevalence occurred on 20 July (13 July, 15 August) (fig. S3), compared with 5 July (30 June, 16 July), as estimated from the routine surveillance data, likely reflecting the rapid increase in testing capacity (fig. S3).

**Fig. 2:**
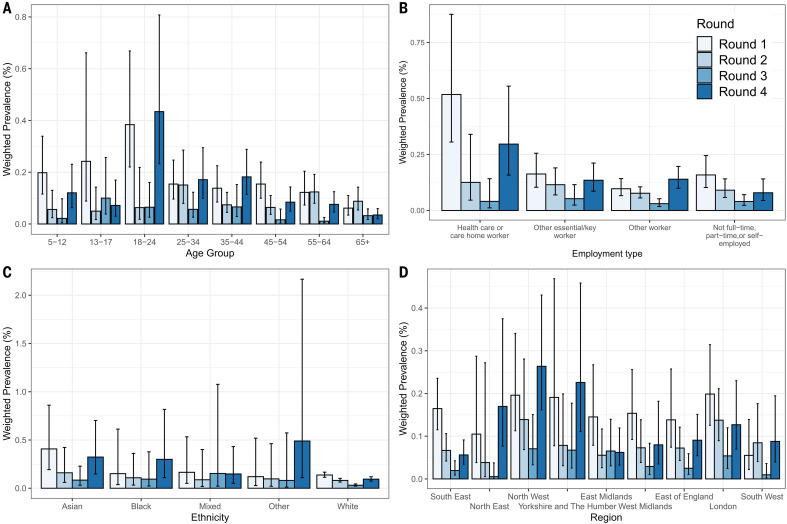
Prevalence of unweighted swab positivity. Covering four rounds of the REACT-1 study by (**A**) age, (**B**) employment type, (**C**) ethnicity, and (**D**) region. Vertical bars show 95% confidence intervals. Rounds are differentiated by color.

During March and April, the highest prevalence regionally was recorded in London, which experienced the highest incidence of cases during the first wave (*21*, *22*). Prevalence fell in all regions between round 1 (1 May to 1 June) and round 3 (24 July to 11 August). There was then positive growth (>95% probability) between round 3 and round 4 (20 August to 8 September) in all regions except East and West Midlands (table S3 and figs. S4 and S5), with the highest growth in the North East region [*R* = 1.67 (1.20, 2.48)]. During round 4 (20 August to 8 September), we observed about a threefold difference between the highest prevalence in both the North West region at 0.17% (0.12%, 0.24%) and Yorkshire and the Humber at 0.17% (0.11%, 0.27%) and the lowest at 0.06% (0.04%, 0.09%) in the South East region (Fig. 2, table S4, and fig. S4).

**Fig. 3:**
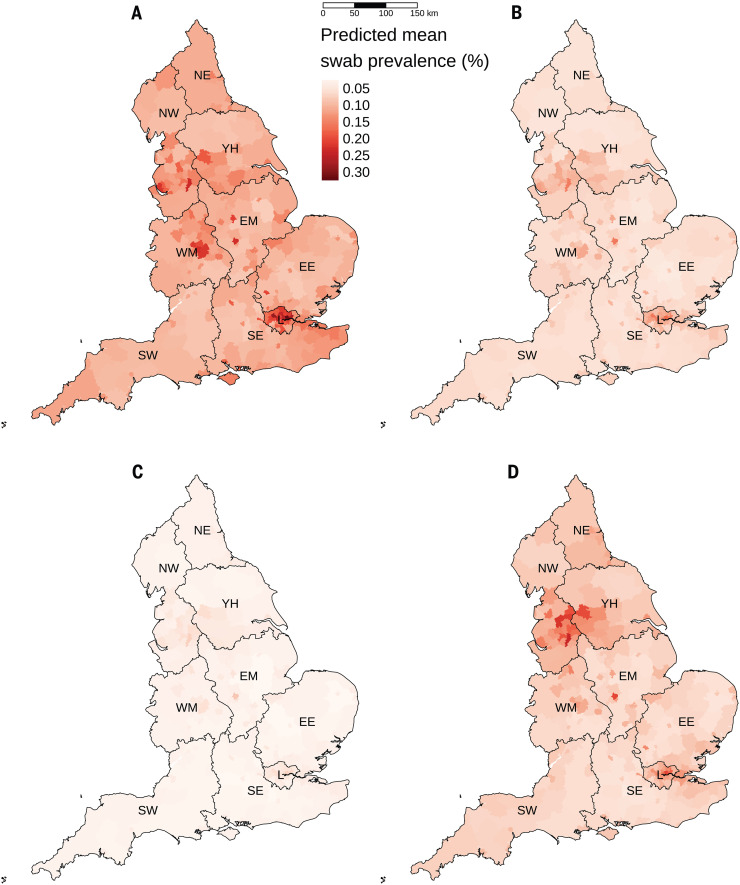
Geospatial patterns. Estimated prevalence from geospatial model for (**A**) round 1, (**B**) round 2, (**C**) round 3, and (**D**) round 4. Regions: NE, North East; NW, North West; YH, Yorkshire and the Humber; EM, East Midlands; WM, West Midlands; EE, East of England; L, London; SE, South East; SW, South West.

We found spatial heterogeneity in prevalence at a subregional level using a geospatial model (*16*) with a range parameter estimate of 22.6 km (95% confidence interval: 16.1, 31.7) (Fig. 3 and table S5). We observed areas of higher prevalence in parts of the North West region, Yorkshire and the Humber, Midlands, and the London conurbation in round 1 (1 May to 1 June). These patterns persisted at lower prevalence in round 2 (19 June to 7 July) before reaching lowest prevalence in round 3 (24 July to 11 August). The epidemic then resurged in round 4 (20 August to 8 September), with geographical patterns similar to those seen in rounds 1 and 2 and an indication that prevalence in each local area had increased between rounds 3 and 4 (fig. S5).

Our findings show substantial variations in age patterns over time. In round 4 (20 August to 8 September), the highest prevalence at 0.25% (0.15%, 0.41%) was found in participants aged 18 to 24 years, increasing more than threefold from 0.08% (0.04%, 0.18%) in round 3 (24 July to 11 August) (Fig. 2 and table S4). The lowest prevalence at 0.04% (0.02%, 0.06%) was in those aged 65 years and older, similar to round 3. These patterns suggest that the second wave started in young adults–likely driven by higher numbers of social contacts (*23*)–before spreading into older (*22*, *24*) and more at-risk populations (*25*).

We compared age patterns from REACT-1 with those in the routine surveillance case incidence data (*17*); in each dataset, we estimated odds ratios for each age group (35 to 44 years as comparator) (fig. S6). We found that the symptomatic case data in round 1 (1 May to 1 June) overestimated odds at older ages and underestimated odds at younger ages relative to REACT-1, reflecting the limited availability of symptomatic testing at that time, when testing was carried out mainly among hospitalized patients (*17*). In subsequent rounds, the case data consistently underestimated odds at ages 5 to 14 years, while odds at older ages continued to be overestimated relative to REACT-1. Similar biases in case data may have contributed to reports of reduced susceptibility to infection in younger children (*26*).

We found differences over time in the odds of infection for health care and care home workers, with odds of 5.5 (3.1, 9.7) relative to other workers during round 1 (1 May to 1 June) but much-reduced odds in subsequent rounds (table S6). These findings indicate that there was a shift away from rapid transmission in hospitals (*27*) and care homes (*28*) during the first wave to predominantly community transmission at the start of the second wave.

We found about a twofold greater unweighted prevalence of swab positivity in participants of Asian ethnicity (mainly South Asian) at 0.14% (0.10%, 0.20%), compared with 0.07% (0.07%, 0.08%) in white participants across all four rounds combined (table S4); odds were 2.2 (1.2, 4.0) relative to white participants in round 4 (20 August to 8 September), with multiple adjustment (table S6). There was also a higher unadjusted prevalence of infection in Black people compared with white people across all four rounds combined at 0.15% (0.09%, 0.27%) (table S4). These higher rates of swab positivity are consistent with higher SARS-CoV-2 seroprevalence among Asian and Black people and people of other nonwhite ethnicity in England (*22*). This supports the view that higher rates of hospitalization and mortality from COVID-19 reported among minority ethnic groups in England (*29*) reflect their higher rates of infection rather than a poorer prognosis once infected.

Although we aimed to be representative of the population of England by inviting a random sample of people on the National Health Service patient register (*16*), we found differential response rates by age, area, and round. For example, response rates ranged from 21.8% in round 4 (20 August to 8 September) to 30.8% in round 1 (1 May to 1 June) and differed across age groups, from 10.7% for ages 18 to 24 years to 31.1% for ages 55 to 64 years (round 4) (table S1). However, unlike the symptomatic testing, we were able to correct for variations in response given that we have a known denominator. We were thus able to estimate prevalence weighted to the population of England as a whole, taking into account sample design and nonresponse, although we did not reweight prevalence estimates for subgroups, because of lower numbers of positives.

We converted growth rates into reproduction numbers using serial interval parameters from (*30*). However, we also tested the sensitivity of our results to a wide range of other published estimates (table S7). We found that by using (*30*) our estimates of *R* above 1 were conservative and that using other published parameters lowered our *R* estimates. The converse was true for *R* values less than 1; estimates using (*30*) were lower than those using results from other studies. Essentially, uncertainty in our estimates of *R* reflect uncertainty in our estimate of the growth rate and do not propagate uncertainty about the serial interval present in the literature.

We relied on self-swabbing to obtain estimates of swab positivity. A throat and nose swab is estimated to have between ~70 and ~80% sensitivity (*31*), so we are likely to have underestimated true prevalence, although this would be unlikely to have affected trend analyses or estimation of *R*. During the period of our study, there was changing availability of symptom-driven test capacity, which likely explains the earlier increase in swab positivity in the symptomatic data compared with our own data (*17*). The trends in our data were supported by results of analyses among the subset of nonsymptomatic individuals, who would not have presented to the national case-testing program (table S2).

Our study provides timely community-based prevalence data to increase situational awareness and inform the public health response during the current SARS-CoV-2 pandemic. The scenario of declining prevalence to low levels followed by resurgence reported here may reoccur in the future in the absence of protective population immunity; this depends on levels of vaccine coverage of the population (*32*), degree of waning of natural immunity and vaccine efficacy (*33*), and potential for antigenic escape (*34*). Also, as of early 2021, some populations have successfully avoided large waves of infection but may not be able to do so in the future because of intervention fatigue or increased transmissibility of the virus (*35*).

Accurate estimates of prevalence with robust descriptions of trends by time, person, and place would support sustainable policies designed to maintain low levels of prevalence. Unlike China, New Zealand, and Australia, the UK did not attempt functional elimination (so-called COVID-zero) during periods of low prevalence in February or August 2020, in common with all other European nations. However, with the rollout of effective vaccines from December 2020 (*36*) and with accumulating evidence of antigenic change (*37*), the cost-benefit assessment of policies designed to achieve sustained low levels of prevalence may be different in the future. For example, during the declining phase, prevalence may be high in some areas because of low vaccine uptake, variant emergence, or increased social mixing. Data from REACT-1 or similar studies could be used to target local public health or vaccination campaigns more effectively than would be possible with routine surveillance data alone, similar to how REACT-1 results fed into the government policy of the rule of six in early September 2020 (*19*).

Additionally, knowledge from community-based surveillance can be used to calibrate other data streams, not only symptomatic testing (*38*) but also the use of mobility data (*39*) and sewage-based sampling of viral RNA (*40*). Given the different spatial and temporal resolutions of alternate data sources, ground-truth data such as those from REACT-1 can substantially improve evidence synthesis for infectious disease (*41*).

We demonstrate the capability of a large national community surveillance program to detect a resurgence of SARS-CoV-2 infection at low prevalence. Our findings have implications for policies to contain the COVID-19 pandemic. While we wait for the vaccination of all risk groups in England and across the world, control of the SARS-CoV-2 virus must continue to rely on established public health measures (*42*), including social distancing, frequent handwashing, face coverings, and an effective test, trace, and isolate system. Although we show high levels of effectiveness of stringent social distancing during the first lockdown in England, prevalence subsequently increased. This perhaps reflects holiday travel, return to work, or a more general increase in the number and transmission potential of social interactions, with a rapid rise evident in early September 2020 at the start of the second wave. A combination of vaccination, social distancing, and other public health measures should again result in substantial reductions in prevalence. Studies similar to REACT-1 could then detect any upturn in prevalence and help trigger an effective public health response.

## Supplementary Materials

science.sciencemag.org/content/372/6545/990/suppl/DC1 Materials and Methods Figs. S1 to S6 Tables S1 to S7 References (*44*–*52*) Data S1 MDAR Reproducibility Checklist

## Appendix

View/request a protocol for this paper from *Bio-protocol*.
